# Left Ventricular Diastolic Function in Subjects Conceived through Assisted Reproductive Technologies

**DOI:** 10.3390/jcm11237128

**Published:** 2022-11-30

**Authors:** Franziska Sciuk, Theresa Vilsmaier, Marie Kramer, Magdalena Langer, Brenda Kolbinger, Pengzhu Li, André Jakob, Nina Rogenhofer, Robert Dalla-Pozza, Christian Thaler, Nikolaus Alexander Haas, Felix Sebastian Oberhoffer

**Affiliations:** 1 Division of Pediatric Cardiology and Intensive Care, University Hospital, LMU Munich, Marchioninistraße 15, 81377 Munich, Germany; 2 Division of Gynecological Endocrinology and Reproductive Medicine, Department of Obstetrics and Gynecology, University Hospital, LMU Munich, Marchioninistraße 15, 81377 Munich, Germany

**Keywords:** assisted reproductive technologies, pediatrics, echocardiography, left ventricular diastolic function, cardiac function

## Abstract

Subjects conceived through assisted reproductive technologies (ART) potentially suffer from impaired left ventricular (LV) function due to premature vascular aging. This study aimed to evaluate whether subtle differences in LV diastolic function can be observed echocardiographically between young ART subjects and their spontaneously conceived peers. The echocardiographic assessment included the measurement of LV dimensions, mitral inflow velocities, and myocardial velocity at early diastole (E’, cm/s) at the LV wall and the interventricular septum (IVS). An average from E/E’LV and E/E’IVS (E/E’AVG) was derived. In total, 66 ART subjects and 83 controls (12.85 ± 5.80 years vs. 13.25 ± 5.89 years, *p* = 0.677) were included. The ART subjects demonstrated a significantly lower E’LV (19.29 ± 3.29 cm/s vs. 20.67 ± 3.78 cm/s, *p* = 0.020) compared to their spontaneously conceived peers. Study participants of ≥ 10 years of age displayed a significantly higher E/E’AVG (6.50 ± 0.97 vs. 6.05 ± 0.99, *p* = 0.035) within the ART cohort. The results of this study demonstrate a significantly lower LV diastolic function in the ART subjects. However, no significant changes in LV diastolic function were observed between the two groups when the results were adjusted for age, birth weight percentile, and gestational age. Those ART subjects born preterm might have an elevated risk of developing LV diastolic alterations and could therefore profit from close echocardiographic monitoring.

## 1. Introduction

Over 186 million individuals worldwide suffer from infertility [1]. The World Health Organization defines infertility as a condition of the male or female reproductive system which results in the nonachievement of clinical pregnancy within twelve months of trying [2]. More than half of couples affected by infertility seek medical treatment [3], including assisted reproductive technologies (ART). Since its first use in 1978, ART has gained remarkable popularity [4]. More than eight million children worldwide have been conceived through ART methods, such as intracytoplasmic sperm injection (ICSI) or in vitro fertilization (IVF) [5]. An increasing number of clinical and experimental studies identified conception through ART as a potential cardiovascular risk factor, putting the long-term health effects of the offspring into question [6]. The underlying mechanisms leading to an altered cardiovascular phenotype have not been fully understood yet. It is suggested that ART-induced epigenetic changes might occur during the vulnerable period of embryonal development [7]. In addition, parental and perinatal risk factors, such as multiple pregnancy, prematurity, or low birth weight, are considered to be of influence [6,8]. Findings of vascular alterations, including elevated arterial stiffness, endothelial dysfunction, and increased carotid intima-media thickness (cIMT), indicate premature vascular aging in ART offspring [6,8,9,10]. Furthermore, some studies describe elevated blood pressure levels in ART subjects [9,11,12], suggesting that arterial hypertension might be one of the first manifestations of premature vascular aging from ART [9]. Hypertension is closely linked with an augmentation of left ventricular (LV) afterload and the onset of LV dysfunction [13]. In hypertensive patients, alterations in LV diastolic function can already be seen before LV systolic dysfunction is detectable [13]. Despite the distinct vascular morbidity observed in ART subjects, limited data on LV function are available [12]. For the evaluation of LV diastolic function, echocardiographic assessment, including Doppler echocardiography and tissue Doppler imaging (TDI), reveals valuable information about LV relaxation, filling dynamics, and compliance [14,15].

This study aimed to evaluate whether subtle differences in LV diastolic function can be observed echocardiographically in young ART subjects compared to their spontaneously conceived peers.

## 2. Materials and Methods

### 2.1. Study Population and Study Design

This study was conducted within the Division of Pediatric Cardiology and Intensive Care, University Hospital, LMU Munich (Munich, Germany), between May 2021 and March 2022. The families that were treated at the Division of Gynecological Endocrinology and Reproductive Medicine, Department of Obstetrics and Gynecology, University Hospital, LMU Munich (Munich, Germany) and whose children were successfully conceived through ART were invited for a cardiovascular examination. Age and sex-matched controls were acquired within the greater Munich (Germany) area via public calls. Only healthy, spontaneously conceived peers without a history of cardiovascular disease were eligible for enrollment. In order to assess the influence of age on cardiovascular health, participants of different developmental stages (children, adolescents, and young adults) were included in this study.

### 2.2. Medical History, Physical Examination, Pregnancy and Birth, and Level of Education

Participants were questioned about their medical history with special emphasis on cardiovascular morbidity (e.g., congenital heart disease, arterial hypertension, sugar metabolism disorders, and lipid metabolism disorders). Information regarding the regular use of medication with potential impact on the cardiovascular system, as well as the smoking status, was gathered. A physical examination was conducted on each study participant. Body weight (kg) and body height (cm) were measured, and the body mass index (BMI, kg/m^2^) was calculated. For minor study participants, weight classification was assessed through BMI percentiles (P.) established by Kromeyer-Hauschild et al. (underweight if BMI < 10 P., normal weight if BMI ≥ 10 P. but < 90 P., overweight if BMI ≥ 90 P. but < 97 P., and obese if BMI ≥ 97 P.) [16]. For subjects ≥ 18 years of age, weight classification was determined by absolute BMI values (underweight if BMI < 18.5 kg/m^2^, normal weight if BMI ≥ 18.5 kg/m^2^ but < 25 kg/m^2^, overweight if BMI ≥ 25 kg/m^2^ but < 30 kg/m^2^, and obese if BMI ≥ 30 kg/m^2^). Body surface area (BSA, m^2^) was calculated based on the Mosteller formula [17]. Systolic blood pressure (SBP, mmHg) and diastolic blood pressure (DBP, mmHg) were assessed using an automated blood pressure measurement device (Connex Spot Monitor, 901058 Vital Signs Monitor Core, Welch Allyn, Inc., Skaneateles Falls, NY, USA). Heart rate (bpm) was recorded during echocardiographic examination through three-lead ECG tracking. In order to determine the levels of the N-terminal prohormone of brain natriuretic peptide (NT-proBNP, pg/mL), blood samples were drawn and analyzed. Data on NT-proBNP were transformed into a decadic log-scale. Information on birth weight (g), gestational age (weeks), multiple pregnancy, maternal age at birth (years), maternal BMI at conception (kg/m^2^), gestational diabetes, and maternal blood pressure during pregnancy ≥ 140/90 mmHg were additionally obtained. The above-mentioned information was assessed by questioning the participant’s parents and by screening clinical records. P. of birth weight was calculated according to Voigt et al. [18]. Maternal educational level was determined according to the German educational system: no school leaving qualification (0), lower secondary school leaving certificate (1), intermediate secondary school leaving certificate (2), general qualification for university entrance (3), completed apprenticeship (4), and completed university degree (5).

### 2.3. Echocardiographic Assessment of Left Ventricular Diastolic Function

Echocardiographic assessment was performed for all study participants by one investigator. Echocardiographic images were acquired using a Philips iE33 xMatrix or a Philips Epiq 7G ultrasound device (Philips Healthcare, Amsterdam, The Netherlands) with a 1–5 MHz or a 3–8 MHz sector ultrasound transducer (Philips Healthcare, Amsterdam, The Netherlands). Echocardiography was performed under constant three-lead ECG tracking. Three consecutive loops were recorded and transferred to an offline workstation for further analysis (IntelliSpace Cardiovascular Ultrasound Viewer, Philips Healthcare, Amsterdam, the Netherlands). The echocardiographic offline analysis was performed by one investigator for all study participants.

#### 2.3.1. Left Ventricular Dimensions

**Figure 1:**
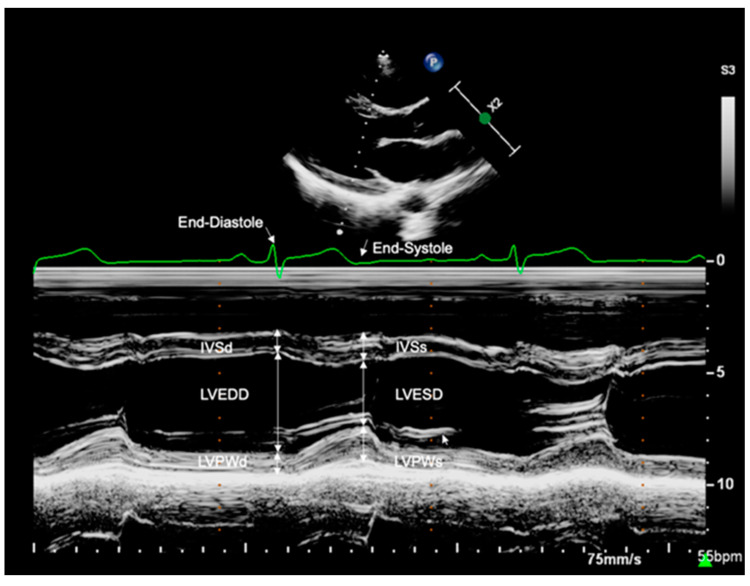
Measurement of left ventricular dimensions. M-Mode echocardiography was performed at the level of the mitral valve tip in parasternal long-axis view. End-diastole (QRS in ECG) and end-systole (end of T-wave in ECG) were determined through simultaneous ECG tracking. LV dimensions were assessed offline. IVSd, interventricular septum thickness at end-diastole; IVSs, interventricular septum thickness at end-systole; LVEDD, left ventricular end-diastolic diameter; LVESD, left ventricular end-systolic diameter; LVPWd, left ventricular posterior wall thickness at end-diastole; LVPWs, left ventricular posterior wall thickness at end-systole.

M-Mode echocardiography was performed at the level of the mitral valve tip in parasternal long-axis view (Figure 1). End-diastole (QRS in ECG) and end-systole (end of T-wave in ECG) were determined through simultaneous ECG tracking. The following LV dimensions were assessed using an offline workstation: interventricular septum thickness at end-diastole (IVSd, mm), interventricular septum thickness at end-systole (IVSs, mm), LV end-diastolic diameter (LVEDD, mm), LV end-systolic diameter (LVESD, mm), LV posterior wall thickness at end-diastole (LVPWd, mm), and LV posterior wall thickness at end-systole (LVPWs, mm). For minor study participants, the z-scores of cardiac structures were calculated according to Kampmann et al. [19].

#### 2.3.2. Mitral Inflow Velocities

**Figure 2:**
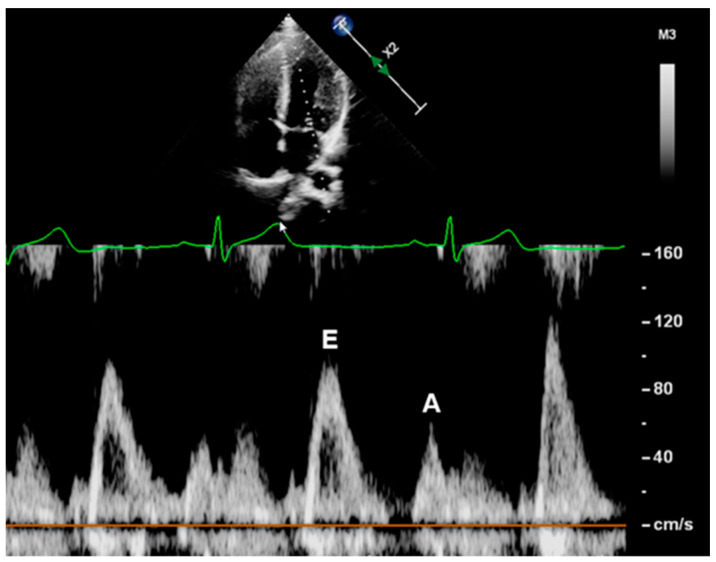
Measurement of mitral inflow velocities. The sample volume of a pulsed-wave Doppler was placed at the tip of the mitral valve in an apical four-chamber view. The mitral peak flow velocities at early (E, cm/s) and late (A, cm/s) diastole were measured offline.

Mitral inflow velocities were derived through pulsed-wave Doppler sonography. The sample volume was placed at the tip of the mitral valve in apical four chamber view. Mitral peak flow velocities of early (E, cm/s) and late (A, cm/s) diastole were measured offline (Figure 2). The contribution of flow between the early and late diastolic filling phases (E/A) was calculated.

#### 2.3.3. Myocardial Peak Velocities

**Figure 3:**
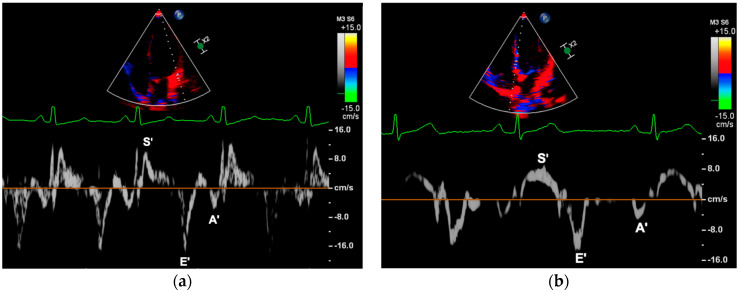
Measurement of myocardial peak velocities; (**a**) left ventricular wall, (**b**) interventricular septum. Images were obtained by placing the sample volume on the lateral and septal mitral annulus in an apical four-chamber view. Myocardial systolic (S’, cm/s), early diastolic (E’, cm/s), and late diastolic (A’, cm/s) peak velocities were measured offline.

TDI was used to obtain myocardial systolic (S’, cm/s), early diastolic (E’, cm/s), and late diastolic (A’, cm/s) peak velocity, measured at the right ventricular wall (RV), interventricular septum (IVS), and LV wall. Images were acquired from an apical four-chamber view by placing the Doppler sample volume on the lateral tricuspid annulus, as well as the septal and lateral mitral annulus (Figure 3). Myocardial peak velocities were determined through offline analysis. Ultimately, the ratios of E/E’LV and E/E’IVS were assessed. An average of E/E’LV and E/E’IVS was calculated (E/E’AVG).

### 2.4. Statistical Analysis

Statistical analysis was performed with SPSS version 27 (IBM SPSS Statistics for Windows, version 27.0. IBM Corp., Armonk, NY, USA). Continuous variables were tested for normal distribution using the Kolmogorov–Smirnov test and Shapiro–Wilk test. Continuous variables with a normal distribution were analyzed using an independent *t*-test and are displayed as mean ± standard deviation (SD). Non-normally distributed continuous variables were analyzed using the Mann–Whitney-U test and are displayed as a median (range). Large sample sizes (*n* ≥ 30) were considered normally distributed and an independent *t*-test was used for analysis. Categorical variables are expressed in counts or percentages and were analyzed using Pearson’s Chi-squared test or Fisher’s exact test. The Pearson correlation coefficient was used to display correlations between normally distributed variables. For the non-normally distributed variables, the Spearman’s rank correlation coefficient was utilized. For the statistical comparison of the correlations, the z-scores were generated using Cocor software (http://comparingcorrelations.org/, accessed on 27 October 2022) [20]. Normal z-score levels were considered to range between −1.96 and 1.96 at a 95% confidence level. Linear regression was utilized to adjust for the influence of age, birth weight P., and gestational age on LV diastolic morphology and LV diastolic function. In order to adjust the alpha level for multiple testing, the Holm–Bonferroni correction was applied. A *p*-value of < 0.05 was considered as significant. In order to elaborate on the influence of age on LV diastolic function, subanalyses for subjects < 10 years of age and subjects ≥ 10 years of age were performed. These age ranges were utilized to distinguish between prepubertal and pubertal/postpubertal subjects as well as enable a sufficient sample size for the respective subanalyses. Moreover, a correlation analysis of the LV diastolic function parameters and age was conducted.

## 3. Results

### 3.1. Patients’ Characteristics

In total, 156 children, adolescents, and young adults were recruited for this study; two ART individuals were excluded from further analysis due to having a medical history of T-cell lymphoma and heart surgery; two ART subjects and three spontaneously conceived peers were excluded due to an incomplete echocardiographic data assessment. Overall, 66 ART subjects (16 IVF, 49 ICSI, and one gamete intrafallopian transfer (GIFT)) and 83 control subjects were considered for the final data analysis. A total of five ART study participants displayed conditions with potential impact on their cardiovascular function, including a bicuspid aortic valve, a questionable history of myocarditis, a history of hypercholesteremia, long QT syndrome, and hypothyroidism. Four of the ART individuals used oral contraceptives, one used L-thyroxine, and one used methylphenidate. In the control group, six of the subjects were taking oral contraceptives, one bisoprolol due to recurrent migraines and another methylphenidate. None of the study participants suffered from cardiac failure and/or cardiac arrest.

The ART and control groups did not differ significantly in age (12.85 ± 5.80 years vs. 13.25 ± 5.89 years, *p* = 0.677) and sex (38 female vs. 43 female, *p* = 0.483). The age of ART study participants ranged between 4 and 24 years; the age of the spontaneously conceived peers ranged between 4 and 26 years. No differences in anthropometric parameters (body weight, body height, BMI, and BSA), weight classification, SBP (*p* = 0.952, adjusted *p* > 0.999), DBP (*p* = 0.843, adjusted *p* > 0.999), heart rate (*p* = 0.695, adjusted *p* > 0.999), and smoking status were detected between the two groups. However, the ART group displayed significantly lower values for birth weight, birth weight P., and gestational age. Further, a significantly higher prevalence of multiple pregnancies and a significantly higher maternal age at birth were shown for the ART subjects. The remaining perinatal and maternal parameters, including maternal educational level, were comparable between both of the groups. Detailed information on the patient’s characteristics is given in Table 1.

### 3.2. Left Ventricular Diastolic Function

Compared to the control group, the ART subjects showed no difference in LV dimensions, mitral inflow velocities, and the ratio of E/A. The TDI analysis displayed a significantly lower E‘LV within the ART cohort. One adolescent in the control group displayed a ratio of E/E’LV ≥ 8. The ratios of E/E’LV, E/E’IVS, and E/E’AVG did not differ significantly between the two groups yet showed an overall tendency to be higher in the ART group. Log (NT-proBNP) showed no significant difference between the two groups. After adjusting for age, birth weight P., gestational age, and correction for multiple testing, no significant differences in LV dimension and diastolic function were displayed between the two groups. Table 2 and Table 3 summarize the detailed information on LV dimensions and diastolic function for the ART and control groups.

When comparing the 24 ART subjects and 59 spontaneously conceived peers (9.53 (5.30–24.33) years vs. 11.95 (4.34–26.05) years, *p* = 0.413; 14 female vs. 28 female, *p* = 0.369) who were born as full term singletons (≥ 37 weeks of gestation, birth weight ≥ 10. P.), no significant differences in E’LV (19.63 ± 3.63 cm/s vs. 21.04 ± 4.03 cm/s, *p* = 0.142, adjusted *p* = 0.552) and the remaining parameters of LV diastolic function were observed between both groups.

### 3.3. Influence of Age

In order to further elaborate on the influence of age on LV diastolic function, study participants of < 10 years of age were analyzed. The subanalysis included 28 ART subjects and 30 spontaneously conceived peers. The ART and control groups did not differ significantly in age (7.37 (4.41–9.82) years vs. 7.33 (4.34–9.84) years, *p* = 0.797) and sex (17 female vs. 15 female, *p* = 0.412). ART subjects demonstrated a significantly lower value for A (43.41 ± 9.48 cm/s vs. 51.50 ± 9.21 cm/s, *p* = 0.002, adjusted *p* = 0.004) and a significantly higher E/A ratio (2.33 ± 0.62 vs. 2.01 ± 0.45, *p* = 0.025, adjusted *p* = 0.036). Further, significantly lower values for the IVSs z-score (−0.27 ± 0.83 vs. 0.20 ± 0.77, *p* = 0.032, adjusted *p* = 0.160) were displayed within the ART cohort. The remaining parameters of LV diastolic function did not significantly deviate between both groups.

**Figure 4:**
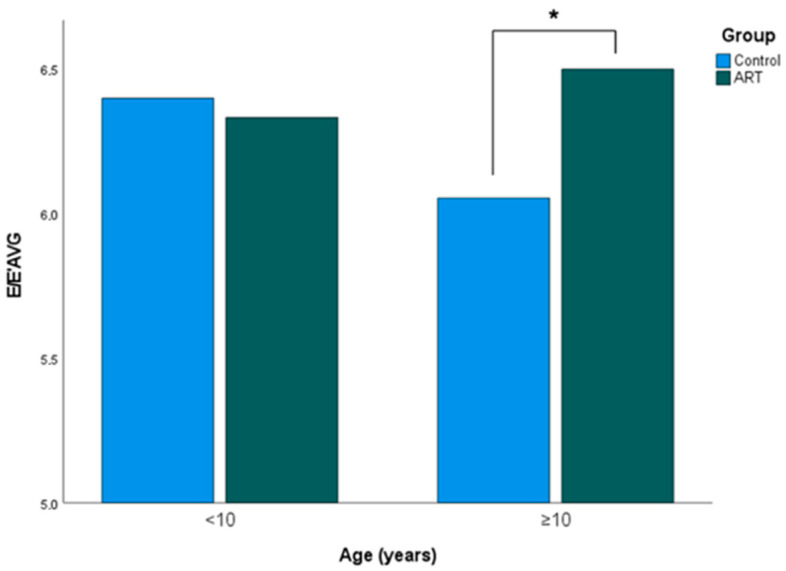
Influence of age on the E/E’AVG ratio in the ART group and control group. The ART subjects (*n* = 28) and spontaneously conceived peers (*n* = 30) of < 10 years of age showed no significant difference in E/E’AVG ratio (6.33 ± 0.77 vs. 6.40 ± 0.88, unadjusted *p* = ns, adjusted *p* = ns). When analyzing subjects ≥ 10 years of age, the ART group (*n* = 38) displayed a significantly higher E/E’AVG ratio (6.50 ± 0.97 vs. 6.05 ± 0.99, unadjusted *p* = 0.035, adjusted *p* > 0.999) compared to the control group (*n* = 53). * *p*-value < 0.05.

Additionally, 38 ART and 53 control subjects ≥ 10 years of age were analyzed. The ART and control groups did not differ significantly in age (16.77 ± 4.46 years vs. 16.54 ± 4.77 years, *p* = 0.814) and sex (21 female vs. 28 female, *p* = 0.818). Interestingly, the ART group displayed a significantly higher E/E’AVG ratio (6.50 ± 0.97 vs. 6.05 ± 0.99, *p* = 0.035, adjusted *p* > 0.999) (Table 4) (Figure 4). The remaining LV diastolic parameters did not differ significantly between both groups (Table 4).

With respect to the variable outcomes within different age groups, a correlation analysis between the LV diastolic function parameters and age was conducted for both groups individually (Table 5). In the control group, age correlated significantly with LVEDD, E’LV, E/E’LV, and E/E’AVG. Within the ART group, age correlated significantly with LVEDD, A, and the E/A ratio. The calculated z-scores indicate a significant difference between both groups for the correlation of A and E/A with age. Interestingly, a significant correlation between SBP and A (r = 0.477, *p* < 0.001), as well as SBP and E/A ratio (r = −0.453, *p* < 0.001), was only shown within the ART group.

## 4. Discussion

This is one of the largest prospective ART studies evaluating cardiac function. In total, 66 ART subjects and 83 spontaneously conceived peers were included. Compared to the control group, those subjects conceived through ART displayed a significantly lower E’LV, indicating a lower myocardial velocity during early diastole. Interestingly, subjects of ≥ 10 years of age revealed a significantly higher E/E’AVG ratio, visualizing a greater LV filling pressure in the ART subjects compared to the spontaneously conceived peers. However, these changes were not observed when the results were adjusted for age, birth weight P., and gestational age.

### 4.1. ART and Cardiovascular Morbidity: Pathophysiological Considerations and a Review of the Literature

#### 4.1.1. Previous Cardiovascular Findings

Growing evidence indicates a connection between ART and adverse cardiovascular conditions. Several cardiovascular alterations, ranging from congenital heart defects to metabolic disorders, were reported in young individuals conceived through ART [6,11,21,22,23]. In 2018, Meister et al. demonstrated that the SBP and DBP levels in ART children were significantly higher than those of the healthy controls. Furthermore, the authors indicated a higher risk of premature vascular aging within the ART group, visualized by a significantly increased cIMT, pulse wave velocity (PWV), and a significantly lower flow-mediated dilation [9]. In conclusion, the authors suggest arterial hypertension to be one of the first clinical manifestations of premature vascular aging in the ART offspring [9].

These findings are consistent with prior studies, underlining the potential association between adverse cardiovascular health and ART [11,22,24]. Ultimately, a meta-analysis, which included 3034 IVF/ICSI and 872 control subjects, demonstrated a significantly higher SBP and DBP (of 1.88 mmHg and 1.51 mmHg, respectively) within the ART cohort [12].

In contrast, several studies could not demonstrate a significantly altered cardiovascular risk profile in ART subjects compared to spontaneously conceived peers [10,25,26,27]. Halliday et al. conducted a large prospective study evaluating blood pressure levels, cIMT, and PWV in 193 ART and 86 control subjects aged 22–35 years [28]. Interestingly, the authors did not find any evidence of increased cardiovascular risk in those subjects conceived through ART. In addition, a recent publication from our department did not reveal significant differences in arterial stiffness between the current ART cohort and spontaneously conceived peers [29].

As arterial hypertension is known to amplify throughout life [30], larger studies evaluating cardiovascular endpoints in ART adults might be required. Still, it is suggested that arterial hypertension is initiated in utero, and premature subjects with a low birth weight are considered to be at specific risk of developing hypertension [30]. Regardless of the pathophysiological origin leading to elevated blood pressure levels, ART subjects—who further display a higher prevalence of being born preterm [6,31]—might benefit from vascular screenings, such as regular ambulatory blood pressure measurements.

Further, it is assumed that cardiovascular alterations in ART subjects affect not only vasculature but also cardiac structure and function [6]. Despite the limited number of studies evaluating cardiac function in ART offspring, higher frequencies of cardiac remodeling, as well as diastolic and systolic dysfunctions, have been reported in the literature [22,26,32,33,34,35,36]. Interestingly, differences in diastolic function seem to be rather consistently observed in ART offspring [12,33,37].

#### 4.1.2. Fetal Programming and Perinatal Risk Factors

The literature suggests that cardiac remodeling is strongly linked to fetal environmental conditions [38]. The structural and functional remodeling of the vasculature and the heart was shown to persist from fetal to postnatal life in ART subjects [22]. Therefore, the early embryonal and fetal stages of life seem to be extremely vulnerable to environmental disturbances [10]. Barker described the hypothesis of “foetal programming of cardiovascular disease”, which explains the crucial role of prenatal growth and the intrauterine environment on the child’s prospective health [39]. Consecutively, prenatal events are presumably linked to the development of chronic disease in adulthood [40].

Periconceptually, the epigenome undergoes multiple changes, potentially leading to increased susceptibility to dysregulations [10,41]. The manipulation of the embryo during ART procedures might perturb epigenetic processes, causing alterations in gene expressions [7]. Additionally, ovarian stimulation and suboptimal culture media have been discussed as influences on the embryo and affecting epigenetic imprinting and perinatal outcomes [42,43]. Interestingly, the increased methylation of the endothelial nitric oxide synthase (eNOS) gene and a subsequently lower expression rate of eNOS were detected in ART mice [44]. Hence, eNOS might potentially be involved in the process of premature vascular aging in ART offspring [44].

The increased prevalence of adverse perinatal conditions linked with ART, such as prematurity, multiple pregnancy, or preeclampsia, should be taken into consideration [6,31]. These perinatal risk factors are assumed to negatively impact cardiovascular health [6]. The literature suggests that the offspring of mothers who suffered from preeclampsia display similar characteristics of cardiovascular alterations as children conceived through ART [45]. Hence, pathological disturbances during fetal life might influence the cardiovascular phenotype as well as long-term health outcomes. Furthermore, parental characteristics, such as elevated maternal age at birth, infertility, or increased cardiovascular morbidity, might alter the cardiovascular phenotype in the ART offspring [6]. Interestingly, experiments in mice without known cardiovascular risk factors demonstrated premature vascular aging as well as arterial hypertension when conceived through ART [44]. Similar results were obtained in ART subjects without the presence of the above-mentioned perinatal risk factors [9,10]. These findings indicate that the procedure of ART itself might be the underlying cause for an altered cardiovascular phenotype.

The current study demonstrated significant differences in LV diastolic function, which were no longer evident after adjustment for age, birth weight P., and gestational age. This suggests that the increased prevalence of adverse perinatal events might substantially contribute to the cardiovascular outcomes of ART subjects.

From a molecular point of view, it is suggested that parental risk factors (e.g., excess weight, diabetes, and smoking), the ART procedure itself (e.g., culture media, fluctuating pH, fluctuating temperature, and a fluctuating oxygen concentration), complications during pregnancy (e.g., hypertensive disorders in pregnancy, gestational diabetes, and intrauterine growth restriction), and adverse perinatal conditions (e.g., prematurity and low birth weight) could increase oxidative stress levels in the offspring [46]. Presumably, higher oxidative stress levels could result in genetic/epigenetic alterations and the later onset of cardiovascular disease within the offspring [46].

To date, the underlying mechanisms causing cardiovascular alterations in ART subjects have not been fully understood. It also remains questionable whether one or the combination of multiple factors leads to higher cardiovascular morbidity in subjects conceived through ART.

Therefore, future studies require a greater sample size for a more precise cardiovascular risk stratification of the ART population. Moreover, molecular studies could help to further understand the link between cardiovascular health and ART.

#### 4.1.3. ART and Left Ventricular Diastolic Function

Regardless of the pathophysiological origins of ART, the resulting cardiovascular impact potentially leads to an altered cardiac phenotype later in life. As illustrated above, arterial hypertension is shown to be a frequent finding in ART subjects and displays one of the greatest risk factors for cardiac dysfunction [47].

The augmentation of blood pressure increases LV afterload and, consequentially, LV wall stress [13,15]. In order to conserve sufficient LV pump function, a compensatory wall thickening occurs, potentially resulting in LV hypertrophy [15]. This cardiac remodeling might lead to an elevation in LV stiffness, often accompanied by a prolonged LV relaxation time [48]. Hence, the deterioration of LV diastolic function can often be assessed before changes in LV systolic function are detectable [13,49]. Another pathological mechanism is associated with arterial hypertension, which affects the regulation of coronary blood flow either by encouraging structural changes in the vasculature or by reducing the responsiveness to mechanical and neurohormonal stimuli [50]. Deficits in myocardial tissue perfusion might lead to cardiac remodeling, which could additionally impair LV diastolic function. This can be observed in patients suffering from coronary artery disease who show signs of impaired relaxation, abnormal diastolic filling patterns, increased muscle stiffness, and attenuated compliance [51,52]. However, in the current study, no differences in SBP, DBP, and LV dimensions between ART subjects and spontaneously conceived peers were observed.

It is further suggested that cardiac remodeling is closely linked with prematurity. Schubert et al. demonstrated that myocardial dysfunction is present in the preterm heart despite normal blood pressure levels and LV mass [53]. Therefore, LV dysfunction in premature subjects might also be caused by intrinsic pathophysiological mechanisms and persists from early life on.

Within the ART group, our findings showed a significant decrease in myocardial velocity E’LV. E’ reflects the myocardial velocity during early diastole, whereas A’ displays the myocardial velocity during late diastole, driven by atrial contraction [54]. Depending on the level of compliance, most of the LV diastolic filling takes place during early diastole and is essentially facilitated by LV relaxation [55]. As E’ is a marker for LV relaxation [56], a relative reduction in filling during early diastole might be present in ART subjects.

We also found that ART subjects of < 10 years of age displayed a significantly higher E/A ratio and significantly lower values for A. This might reflect a lower contribution of late diastole to the process of diastolic filling. Looking at the ART subjects of ≥ 10 years of age, the E/A ratio and A displayed no significant differences compared to the spontaneously conceived peers. However, this ART subgroup demonstrated a tendency of lower values for E’IVS and E’LV and significantly higher ratios for E/E’AVG, suggesting a potential disturbance in LV relaxation. Moreover, an increase in E/E’AVG might reflect elevated LV filling pressures due to abnormal relaxation patterns [57]. Hence, E/E’AVG can further be used for the estimation of myocardial compliance at end-diastole [58]. The significantly increased E/E’AVG ratio within the ART subjects of ≥ 10 years of age might be compensated for through an increase in atrial pressure over the life span, visualized by the relative elevation in A. Potentially, LV diastolic function might decrease more profoundly with age in ART subjects. However, as the current study did not apply a longitudinal study design, the influence of age on LV diastolic function in ART needs to be interpreted with caution.

Ultimately, it should be noted that LV diastolic function was within the normal range in the majority of our study participants. Only one spontaneously conceived peer displayed a E/E’LV ≥ 8. Moreover, LV dimensions, RV function, and the remaining LV diastolic function parameters did not display significant differences between both groups. Therefore, the clinical relevance of the demonstrated data must be further investigated. Moreover, after the adjustment for age, birth weight P., and gestational age, LV function did not differ significantly between the ART subjects and the spontaneously conceived peers. This was the case for the overall cohort as well as for those study participants of ≥ 10 years of age.

Modifications to the preterm myocardium, which can be observed through different developmental stages, could explain the greater susceptibility of early heart failure in this cohort [59]. However, altered cardiovascular function is reported both for preterm subjects as well as for ART subjects born full-term [9,10,53]. Regardless of the pathophysiological origin of cardiovascular morbidity related to ART, those ART subjects born preterm might display an elevated risk of developing LV diastolic alterations and could therefore profit from close echocardiographic monitoring as a preventive measure. In addition, elevated vascular morbidity—as potentially associated with ART—is closely linked with LV diastolic dysfunction, which is why the regular vascular screening of ART subjects might be beneficial for cardiovascular risk reduction. Ultimately, larger multicenter studies are required to evaluate LV diastolic function in ART subjects longitudinally, enabling precise risk stratification.

### 4.2. Strengths and Limitations

#### 4.2.1. Study Design

This is one of the largest prospective studies evaluating cardiac function in ART subjects. This study included pediatric as well as adult study participants, allowing for the assessment of LV diastolic function during the developmental stages of childhood, adolescence, and adulthood. While care was taken to match the study participants by age and sex, differences in certain lifestyle factors and socioeconomic backgrounds could have potentially influenced the results presented in this study. Furthermore, the ART subjects displayed a significantly higher prevalence of multiple pregnancies and prematurity, and hence a significantly lower birth weight compared to spontaneously conceived peers. In this study, the ART study participants were included regardless of their perinatal risk factors. A prior exclusion of these subjects was thought to potentially distort the presented data and would have resulted in a distinctively smaller sample size of this single-center study.

Data on maternal and perinatal conditions were gathered retrospectively by questioning the parents and by screening clinical records, which led to a certain loss of information.

In this study, we considered GIFT as a conventional form of ART. However, it should be noted that, compared to IVF or ICSI procedures, GIFT does not involve in vitro cultivation, as fertilization takes place in the natural milieu of the fallopian tubes.

The continuous technical development of ART procedures might have had an influence on the cardiovascular outcome of the ART offspring. This limitation needs to be addressed as study participants of different age groups were included in the current study. Moreover, the cardiovascular morbidity of the ART offspring might aggravate during adulthood, leading to increased mortality [60].

Epigenetic changes, prenatal risk factors, and vascular dysfunction may all play a role in the underlying mechanisms that cause cardiovascular changes in ART individuals. Hence, molecular studies could help to further understand the link between cardiovascular health and ART. In the future, larger multicenter studies with a longitudinal study design are required for precise cardiovascular risk stratification in adult ART subjects.

#### 4.2.2. Methodology

In this study, conventional echocardiographic methodologies (e.g., M-Mode, pulsed-wave Doppler, and TDI) were applied to evaluate LV diastolic function. However, novel echocardiographic indices for LV relaxation, LV filling pressure, and LA filling pressure were recently introduced [61]. Such indices include the measurement of LV global longitudinal strain, LV global longitudinal diastolic strain rate, and left atrial strain [61]. Moreover, cardiac magnetic resonance imaging can be considered the gold standard for the assessment of LV function and LV volumes [62]. Therefore, the above-mentioned imaging methodologies should be used in future study designs as they could potentially reveal novel insights into the cardiac function of ART offspring.

## 5. Conclusions

The results of this study suggest a significantly lower LV diastolic function in subjects conceived through ART in comparison to their spontaneously conceived peers. As the only differences demonstrated were within a normal reference range, the clinical importance of these findings has to be further assessed. In addition, LV diastolic function did not differ between both groups when adjusted for age, birth weight P., and gestational age. Potentially, ART subjects born preterm might have an elevated risk of developing LV diastolic dysfunction and could therefore profit from close echocardiographic monitoring. In the future, multicenter studies with a longitudinal design are required for the precise cardiovascular risk stratification of the ART cohort.

## Figures and Tables

**Table 1:** Patients’ characteristics.

Variable	Control (*n* = 83)	ART (*n* = 66)	*p*-Value
Age (years)	13.25 ± 5.89	12.85 ± 5.80	0.677
Female (*n* (%))	43 (51.81)	38 (57.58)	0.483
Body weight (kg)	44.68 ± 18.22	42.37 ± 19.29	0.454
Body height (cm)	151.92 ± 22.67	147.47 ± 21.49	0.225
BMI (kg/m^2^)	18.34 ± 3.26	18.27 ± 3.64	0.898
Weight classification			1.000
*Underweight (n (%))*	6 (7.23)	4 (6.06)	
*Normal weight (n (%))*	71 (85.54)	57 (86.36)	
*Overweight (n (%))*	6 (7.23)	5 (7.58)	
*Obese (n (%))*	0 (0)	0 (0)	
BSA (m^2^)	1.36 ± 0.38	1.30 ± 0.39	0.357
SBP (mmHg)	111.04 ± 11.33	110.92 ± 11.38	0.952
DPB (mmHg)	67.55 ± 7.73	67.82 ± 8.45	0.843
Heart rate (bpm)	72.60 ± 13.13	71.77 ± 12.34	0.695
Smoking (*n* (%))	2 (2.41)	3 (4.55)	0.655
Birth weight (g) ^1^	3393 ± 511	2786 ± 795	<0.001 ***
Birth weight percentile ^2^	52.40 ± 23.80	38.75 ± 24.46	0.001 ***
Gestational age (weeks) ^3^	38.89 ± 1.81	36.84 ± 3.89	<0.001 ***
Multiple pregnancy (*n* (%)) ^4^	3 (4.23)	25 (43.86)	<0.001 ***
Maternal age at birth (years) ^5^	33.15 ± 4.10	35.37 ± 3.75	<0.001 ***
Maternal BMI at conception (kg/m^2^) ^6^	22.03 ± 2.99	23.16 ± 4.26	0.130
Gestational diabetes (*n* (%)) ^7^	3 (4.29)	3 (5.36)	1.000
Maternal blood pressure duringpregnancy ≥ 140/90 mmHg (*n* (%)) ^8^	3 (6.67)	0 (0)	0.281
Maternal educational level ^9^	5 (0–6)	4 (1–5)	0.207

ART, assisted reproductive technologies; BMI, body mass index; BSA, body surface area; SBP, systolic blood pressure; DBP, diastolic blood pressure. Maternal level of education was assessed according to the German educational system: no school leaving qualification (0), lower secondary school leaving certificate (1), intermediate secondary school leaving certificate (2), general qualification for university entrance (3), completed apprenticeship (4), and completed university degree (5). Data are presented as mean ± SD if normally distributed. Categorical data are presented as *n* (%). *** *p*-value ≤ 0.001. A total of ^1^ 77 controls and 64 ART subjects were included in the analysis. A total of ^2^ 75 controls and 61 ART subjects were included in the analysis. A total of ^3^ 75 controls and 61 ART subjects were included in the analysis. A total of ^4^ 71 controls and 57 ART subjects were included in the analysis. A total of ^5^ 81 controls and 65 ART subjects were included in the analysis. A total of ^6^ 59 controls and 46 ART subjects were included in the analysis. A total of ^7^ 70 controls and 56 ART subjects were included in the analysis. A total of ^8^ 45 controls and 28 ART subjects were included in the analysis. A total of ^9^ 52 controls and 45 ART subjects were included in the analysis.

**Table 2:** Left ventricular dimensions.

***Study participants < 18 years of age***				
**Variable**	**Control (*n* = 63)**	**ART (*n* = 49)**	**Unadjusted *p*-value**	**Adjusted *p*-value ^a,b^**
IVSd z-score	0.21 ± 1.02	0.24 ± 1.02	0.857	>0.999
IVSs z-score	0.32 ± 0.78	0.10 ± 0.94	0.179	0.774
LVEDD z-score	−0.43 ± 1.01	−0.34 ± 1.05	0.628	>0.999
LVESD z-score	−0.24 ± 0.90	0.03 ± 0.91	0.130	>0.999
LVPWd z-score	0.27 ± 0.82	0.46 ± 0.82	0.226	0.327
LVPWs z-score	−0.21 ± 0.95	−0.49 ± 0.92	0.118	0.771
***Study participants ≥ 18 years of age***				
**Variable**	**Control (*n* = 20)**	**ART (*n* = 17)**	**Unadjusted *p*-value**	**Adjusted *p*-value ^a,b^**
IVSd (mm)	9.45 (6.70–12.00)	9.00 (7.30–10.00)	0.218	0.761
IVSs (mm)	12.22 ± 1.80	12.04 ± 1.39	0.739	0.921
LVEDD (mm)	45.70 ± 4.11	44.41 ± 2.96	0.289	0.714
LVESD (mm)	30.00 (25.00–35.00)	29.00 (26.00–34.00)	0.469	0.500
LVPWd (mm)	8.70 (6.90–13.00)	8.90 (7.40–11.00)	0.551	0.824
LVPWs (mm)	12.50 (9.80–17.00)	12.00 (9.80–16.00)	0.468	>0.999

ART, assisted reproductive technologies; IVSd, interventricular septum thickness at end-diastole; IVSs, interventricular septum thickness at end-systole; LVEDD, left ventricular end-diastolic diameter; LVESD, left ventricular end-systolic diameter; LVPWd, left ventricular posterior wall thickness at end-diastole; LVPWs, left ventricular posterior wall thickness at end-systole. Data are presented as mean ± SD if normally distributed or median (range) if non-normally distributed. ^a^ Adjusted for age, birth weight percentile, and gestational age. ^b^ Corrected for multiple testing (Holm-Bonferroni).

**Table 3:** Left ventricular diastolic function.

Variable	Control (*n* = 83)	ART (*n* = 66)	Unadjusted *p*-Value	Adjusted *p*-Value ^a,b^
***Mitral Inflow Velocities***				
E (cm/s)	99.82 ± 13.86	98.91 ± 15.46	0.703	>0.999
A (cm/s)	50.44 ± 10.48	47.87 ± 10.83	0.145	0.225
E/A	2.05 ± 0.48	2.16 ± 0.54	0.220	0.297
***Tissue Doppler Imaging***				
S’RV (cm/s) ^1^	12.27 ± 1.78	12.67 ± 2.09	0.222	0.261
E’RV (cm/s) ^2^	14.51 ± 2.92	14.62 ± 2.77	0.833	>0.999
A’RV (cm/s) ^3^	8.31 ± 2.09	8.63 ± 3.20	0.511	0.774
S’LV (cm/s) ^4^	11.48 ± 2.33	10.99 ± 2.84	0.253	>0.999
E’LV (cm/s)	20.67 ± 3.78	19.29 ± 3.29	0.020 *	0.552
A’LV (cm/s)	7.68 ± 2.47	7.05 ± 2.00	0.095	0.894
S’IVS (cm/s)	7.62 ± 1.10	7.61 ± 1.17	0.972	>0.999
E’IVS (cm/s)	13.68 ± 1.77	13.14 ± 1.93	0.076	>0.999
A’IVS (cm/s) ^5^	5.45 ± 1.25	5.03 ± 1.32	0.057	0.312
E/E’LV	4.96 ± 1.01	5.22 ± 0.95	0.099	>0.999
E/E’IVS	7.40 ± 1.30	7.63 ± 1.28	0.279	>0.999
E/E’AVG	6.18 ± 0.96	6.43 ± 0.89	0.105	>0.999
***Blood work***				
Log (NT-proBNP) ^6^	1.56 ± 0.38	1.59 ± 0.34	0.655	>0.999

ART, assisted reproductive technologies; E, mitral peak flow velocity at early diastole; A, mitral peak flow velocity at late diastole; S’, myocardial systolic peak velocity; E’, myocardial early diastolic peak velocity; A’, myocardial late diastolic peak velocity; RV, right ventricle; LV, left ventricle; IVS, interventricular septum; AVG, average; E’AVG = ((E’LV + E’IVS)/2); NT-proBNP, N-terminal prohormone of brain natriuretic peptide. Log refers to the decadic logarithm. Data are presented as mean ± SD if normally distributed. * *p*-value < 0.05. A total of ^1^ 81 controls and 61 ART subjects were included in the analysis. A total of ^2^ 80 controls and 61 ART subjects were included in the analysis. A total of ^3^ 78 controls and 59 ART subjects were included in the analysis. A total of ^4^ 82 controls were included in the analysis. A total of ^5^ 82 controls and 63 ART subjects were included in the analysis. A total of ^6^ 80 controls and 64 ART subjects were included in the analysis. ^a^ Adjusted for age, birth weight percentile, and gestational age. ^b^ Corrected for multiple testing (Holm-Bonferroni).

**Table 4:** Left ventricular diastolic function in Subjects ≥ 10 years of age.

Variable	Control (*n* = 53)	ART (*n* = 38)	Unadjusted *p*-Value	Adjusted *p*-Value ^a,b^
E (cm/s)	99.49 ± 14.32	100.69 ± 17.43	0.719	>0.999
A (cm/s)	49.84 ± 11.18	51.16 ± 10.68	0.575	0.782
E/A	2.08 ± 0.50	2.03 ± 0.44	0.601	0.879
E’LV (cm/s)	21.27 ± 4.00	19.80 ± 3.39	0.068	0.632
E’IVS (cm/s)	13.88 ± 1.98	13.10 ± 2.27	0.084	>0.999
E/E’LV	4.82 ± 1.10	5.18 ± 0.99	0.114	>0.999
E/E’IVS	7.28 ± 1.33	7.82 ± 1.39	0.068	>0.999
E/E’AVG	6.05 ± 0.99	6.50 ± 0.97	0.035 *	>0.999

ART, assisted reproductive technologies; E, mitral peak flow velocity at early diastole; A, mitral peak flow velocity at late diastole; E’, myocardial early diastolic peak velocity; LV, left ventricle; IVS, interventricular septum; AVG, average; E’AVG = ((E’LV + E’IVS)/2). Data are presented as mean ± SD if normally distributed. * *p*-value < 0.05. ^a^ Adjusted for age, birth weight percentile, and gestational age. ^b^ Corrected for multiple testing (Holm-Bonferroni).

**Table 5:** Correlation analysis between age and left ventricular diastolic function.

Variable	Control (*n* = 83)		ART (*n* = 66)		Z_obs_
	r	*p*-Value	r	*p*-Value	
LVEDD (mm)	0.701	<0.001 ***	0.740	<0.001 ***	−0.4822
E (cm/s)	−0.132	0.235	−0.006	0.961	−0.7526
A (cm/s)	−0.079	0.479	0.456	<0.001 ***	−3.3923
E/A	0.030	0.786	−0.427	<0.001 ***	2.8866
E’LV (cm/s)	0.248	0.024 *	0.123	0.327	0.7697
E’IVS (cm/s)	0.067	0.546	−0.071	0.574	0.8206
E/E’LV	−0.346	0.001 ***	−0.164	0.188	−1.1600
E/E’IVS	−0.128	0.248	0.059	0.640	−1.1148
E/E’AVG	−0.236	0.032 *	−0.016	0.899	−1.3330

ART, assisted reproductive technologies; Z_obs_, observed z-score; LVEDD, left ventricular end-diastolic diameter; E, mitral peak flow velocity at early diastole; A, mitral peak flow velocity at late diastole; E’, myocardial early diastolic peak velocity; LV, left ventricle; IVS, interventricular septum; AVG, average; E’AVG = ((E’LV + E’IVS)/2). * *p*-value < 0.05. *** *p*-value ≤ 0.001.

## Data Availability

Not applicable.
